# Supplementary material for: Boom-bust population dynamics increase diversity in evolving competitive communities
Source: Commun Biol. 2021 Apr 23;4:502. doi: 10.1038/s42003-021-02021-4 (PMC8065032; doi:10.1038/s42003-021-02021-4)
Supplement: Supplementary file 2 — Supplementary Information [file 42003_2021_2021_MOESM2_ESM.pdf]

Supplementary Material: Boom-bust population dynamics increase diversity in evolving competitive communities

Michael Doebeli*, Eduardo Cancino Jaque**, Iaroslav Ispolatov**

* Department of Zoology and Department of Mathematics, University of British Columbia, 6270 University Boulevard, Vancouver B.C. Canada, V6T 1Z4; doebeli@zoology.ubc.ca

** Departamento de Física, Universidad de Santiago de Chile, Santiago, Chile, Casilla 302, Correo 2, Santiago, Chile

March 17, 2021

Spontaneous desynchronization of coupled boom-bust populations

Here we present analytical arguments to illustrate that for large β and populations with sufficiently distinct phenotypes, and thus only moderate mutual competitive effect, the boom-bust cycles inevitably desynchronize.

Consider two phenotypes x_1 and x_2 with population sizes N_1 and N_2 whose phenotypes symmetrically diverged from the maximum of the carrying capacity, so that $x_1 = -x_2 \equiv x$. Thus the carrying capacity for each species is equal, $K(x_1) = K(x_2) \equiv K$. The population dynamics of the first species is defined as

$$N_1(t+1) = \frac{\lambda N_1(t)}{1 + (\lambda - 1)\left\{[N_1(t) + \alpha N_2(t)]/K\right\}^{\beta}}, \tag{S1}$$

where $\alpha \equiv \alpha(x_1, x_2)$ is the competitive effect that the two species have on each other (recall that α, given by (4) in the main text, is assumed to be symmetric). The population dynamics of the second species is defined analogously.

For argument's sake, we assume that initially N_1 and N_2 are small, and the second population is slightly larger than the first one, $N_1(0) \equiv N$, $N_2(0) = N + \Delta N$. During the boom phase, the effective population size experienced by phenotype x_1, $N_1(t) + \alpha N_2(t)$, is (much) less than one, so that when elevated to the large power $\beta \gg 1$, this term becomes negligible compared to 1. Then the denominator of (S1) is very close to one. The same is true for N_2, so that both populations grow approximately exponentially,

$$N_1(t) \approx \lambda^t N \tag{S2}$$
$$N_2(t) \approx \lambda^t (N + \Delta N).$$

We assume that the initial difference between the two population sizes is small enough for both of them to crash in the same time step. The populations of two species immediately after the crash step T are

$$N_1(T+1) = \frac{\lambda^{T+1} N}{1 + (\lambda - 1)\left(\frac{\lambda^T [N + \alpha(N + \Delta N)]}{K}\right)^\beta}, \tag{S3}$$
$$N_2(T+1) = \frac{\lambda^{T+1} (N + \Delta N)}{1 + (\lambda - 1)\left(\frac{\lambda^T [N + \Delta N + \alpha N]}{K}\right)^\beta}.$$

We introduce the notation

$$C(t) \equiv \frac{N_1(t) + \alpha N_2(t)}{K}, \tag{S4}$$

and ignore the summand 1 in the denominators of (S3), as at the crash step $C(T)$ exceeds 1 (for the first time in the cycle), so that for $\beta \gg 1$, the second term in the denominators becomes very large, $C^\beta(T) \gg 1$. Thus:

$$N_1(T+1) \approx \frac{\lambda^{T+1} N}{(\lambda-1) C^{\beta}(T)}, \tag{S5}$$

$$N_2(T+1) \approx \frac{\lambda^{T+1}(N+\Delta N)}{(\lambda-1)\left[C(T)+\frac{(1-\alpha)\lambda^T \Delta N}{K}\right]^{\beta}} =$$

$$= \frac{\lambda^{T+1} N}{(\lambda-1) C^{\beta}(T)} \times \frac{1+\frac{\Delta N}{N}}{\left[1+\frac{(1-\alpha)\lambda^T \Delta N}{K C(T)}\right]^{\beta}}.$$

Expanding expression (S5) for $N_2(T+1)$ for small ΔN and observing that the first fraction in this expression is $N_1(T+1)$ and $C(T) \approx \lambda^T N(1+\alpha)/K$, we get

$$N_2(T+1) \approx N_1(T+1)\left\{1 - \frac{\Delta N}{N}\left[\beta\frac{1-\alpha}{1+\alpha} - 1\right]\right\}. \tag{S6}$$

For $\beta \gg 1$ and α noticeably less than 1, the factor in square brackets in (S6) is larger than 1, so that the difference between the two populations N_1 and N_2 after the bust will change sign, $N_2(T+1) < N_1(T+1)$, and will grow in absolute value. Thus, after several boom-bust cycles, the difference $|N_1 - N_2|$ becomes large enough for the minority population to avoid crashing at the same time step as the majority population, and hence to delay its crash by at least one step, as it is illustrated in Fig. S.1.

In fact, the lag between the two populations increases until reaching approximately half the boom-bust period, thus rendering the two populations maximally anti-correlated. This can be seen by assuming that in each crash the population is reduced to the initial population

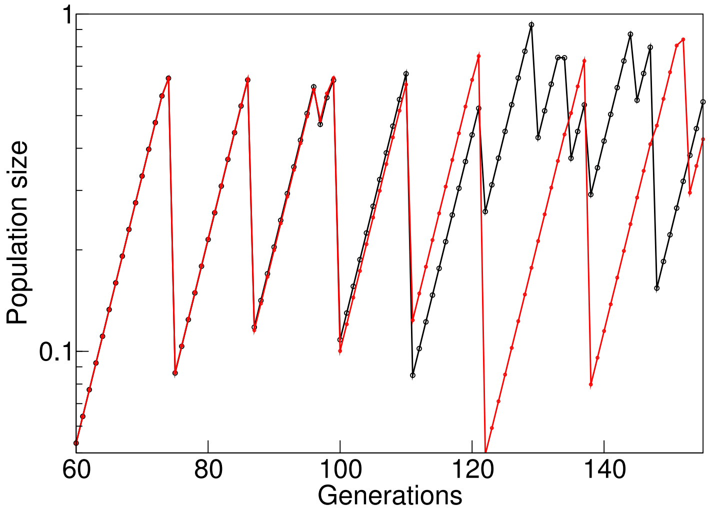

Figure S.1: Illustration of the desynchronization of two populations described by (S5) with initially very similar population sizes. Parameter values $\beta = 40$ and $\alpha = 0.7$.

size N. This is an approximation that ignores the chaotic nature of the population dynamics, but serves to demonstrate the general trend. We denote the initial lag between the first and the second population crashes as Δt steps. Since the first population crashed and resumed its growth earlier, its next crash will come no later than that of the second population. It will occur at step T, where T is the smallest integer that satisfies

$$N\lambda^T \left(1 + \frac{\alpha}{\lambda^{\Delta t}}\right) \geq K. \tag{S7}$$

Here we have taken into account that the second population at time T is $N\lambda^{T-\Delta t}$. After the first population crashes down to N, the second population will continue growing for Δs more steps and will crash at the smallest Δs satisfying the condition

$$N\lambda^{T-\Delta t+\Delta s}\left(1+\alpha\lambda^{\Delta s}\right) = N\lambda^{T-\Delta t+\Delta s}\left(1+\frac{\alpha}{\lambda^{T-\Delta t}}\right) \geq K. \tag{S8}$$

If $T-\Delta t > \Delta t$ (which means $\Delta t < T/2$), that is, if the initial lag between crashes is smaller than half the cycle length, the two conditions (S7, S8) can be simultaneously satisfied only when $\Delta s > \Delta t$, i.e. when the lag between crashes increases. The lag stops increasing when $\Delta t = T/2$, i.e., when the boom-bust cycles of the two populations become maximally desynchronized.

In a general sense, the anti-synchronization of boom-bust cycles can be thought of as the opposite of the well-analyzed phenomenon of synchronization of biological oscillators[1]. The simplest explanation for this disparity are the opposite convexities of growth functions used in these models.

Effect of the extinction threshold on diversity

To show that the reduction in diversity for very large $\beta > 60$ is indeed caused by prevalence of cluster extinction over diversification, we performed simulations varying the extinction threshold. Fig. S.2 (and accompanying videos) shows the evolving community for extinction thresholds 10^{-12} (used for figures in the main text), 10^{-14} and 10^{-10}. Three snapshots of the evolving system at their steady state diversity are shown, illustrating that changing the extinction threshold shifts the balance between extinction and diversification: lower thresholds increase the level of diversity, and with high enough thresholds (and high enough β) diversity is reduced to a single species.

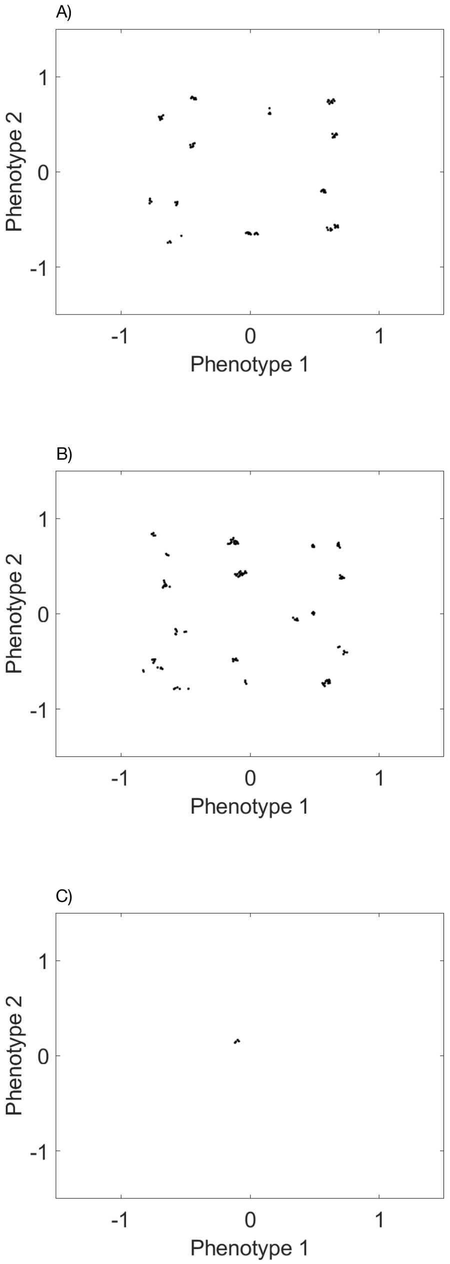

Figure S.2: Snapshots of phenotype distributions after 10^7 generations for three values of the extinction threshold: A) extinction threshold 10^{-12} used for figures in main text; B) lower extinction threshold 10^{-14}; C) higher extinction threshold 10^{-10}. All three scenarios are simulated with very strong overcompensation, $\beta = 110$, and $\lambda = 1.2$. Dimension of phenotype space is $d = 2$, and $\sigma_\alpha = 0.5$. The videos of the evolutionary diversification processes that generated these configurations can be found at figshare.com/s/f2d8ecf480fa372319e1.

Effect of the mutation rate on diversity

Figure S.3A shows the number of species as a function of time (in generations) for different mutation rates (other parameters are as for Figure 2B in the main text). The lower the mutation rate, the longer it takes for diversity to reach saturation levels. Figure S.3B illustrates that the extinction threshold has an effect on the saturation levels of diversity: lower thresholds (fewer extinctions) lead to elevated levels of diversity. For Figure S.3B, the mutation rate was $\mu = 0.005$, so that one mutation of typical size less than 1% of the parental phenotype occurs every 200 generations in the entire evolving community.

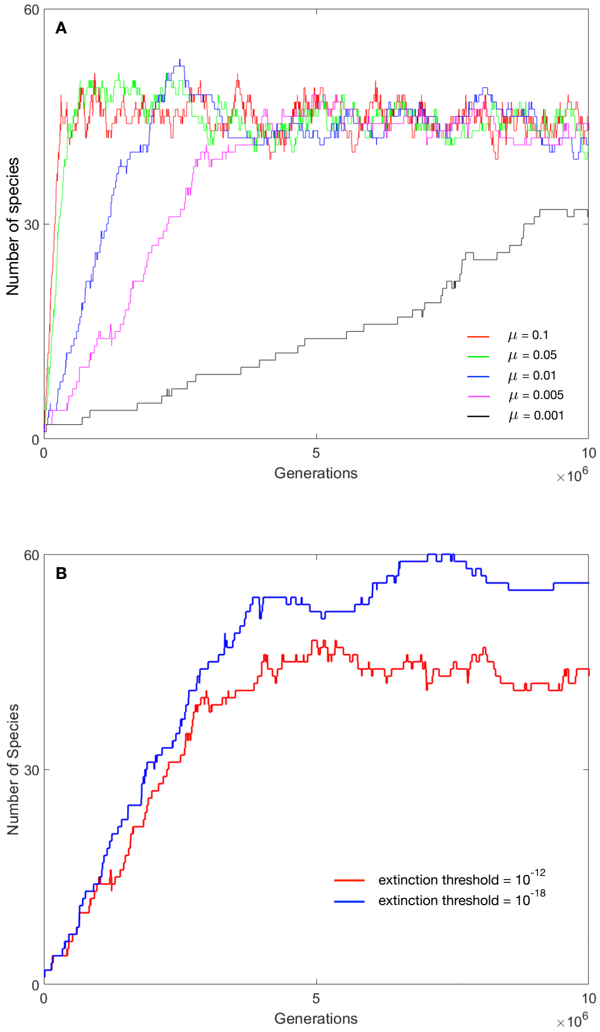

Figure S.3: A: Number of species in the community as a function of time for different mutation rates μ. Species were counted using the clustering algorithm described in the Model and simulation methods section. Other parameter values were as for Fig. 2B in the main text. B: Number of species in the community as a function of time for two different extinction thresholds and for a mutation rate $\mu = 0.005$. Other parameter values were as for Fig. 2B in the main text. For the two scenarios shown in B, videos of the evolutionary process can be found at figshare.com/s/f2d8ecf480fa372319e1.

References

[1] Mirollo, R.E. & Strogatz, S.H. (1990). Synchronization of pulse-coupled biological oscillators. *SIAM Journal on Applied Mathematics*, 50, 1645–1662.
